# Editorial: Regulatory T Cell Heterogeneity: Canonical and Non-Canonical Functions

**DOI:** 10.3389/fimmu.2021.722563

**Published:** 2021-09-22

**Authors:** Esen Sefik, Shohei Hori, Ajithkumar Vasanthakumar

**Affiliations:** ^1^Immunobiology, Yale University School of Medicine, New Haven, CT, United States; ^2^Graduate School of Pharmaceutical Sciences, The University of Tokyo, Bunkyō, Japan; ^3^Department of Microbiology and Immunology, Peter Doherty Institute for Infection and Immunity, Melbourne, VIC, Australia; ^4^Cancer Immunobiology Program, Olivia Newton-John Cancer Research Institute, Melbourne, VIC, Australia

**Keywords:** regulatory T cells, Foxp3, tissue homeostasis, autoimmunity, gut immunity

Foxp3+ Regulatory T (Treg) cells suppress self-reactive T cells and inflammatory immune cells to protect from autoimmunity and excessive inflammation (1). Treg cells therefore are critical to preserving immune homeostasis (1). Over the last decade however, several landmark studies have revealed the multifaceted role of Treg cells, many that extend beyond the immune system (2, 3). Notably, they regulate multiple non-immune functions that include tissue repair, organismal metabolism, and neuronal pruning, highlighting the importance of Treg cells in preserving tissue homeostasis (3). Diverse functions of Treg cells are accompanied by diverse origins and tissue locations. While most Treg cells develop in the thymus, conventional CD4+ T cells can also upregulate the lineage specific transcription factor Foxp3 to become Treg cells outside of the thymus in response to antigenic exposure in environmental interfaces such as the gastrointestinal tract (4). Developmentally, therefore, Treg cells are classified as thymic (tTreg) and peripherally derived (pTreg) and utilize distinct molecular pathways to commit to the Treg cell lineage. While both Treg cell types need T cell receptor (TCR) and IL-2 signaling for their development and maintenance, pTreg cells require additional signals such as TGF-β to commit to the Treg cell linegae (1). Once developed in the thymus, Treg cells migrate to secondary lymphoid tissues and non-lymphoid tissues, where they have additional tissue specific homeostatic requirements (3). Tissue specific expression of unique transcription factors and cytokine receptors are imperative for the differentiation, maintenance, and function of Treg cells in several non-lymphoid tissues (3) (e.g. intestines, visceral adipose tissue (VAT), muscle, and brain). Transcription factors such as PPAR-*γ*, ROR*γ*t, and c-Maf as well as the cytokine IL-33 are a few of the known tissue tropic factors in the Treg cell lineage (4–7). Notably, ablation of these factors impairs Treg cell populations and their functions specifically within the tissues they are lodged in. For example, ablation of PPAR-*γ* or IL-33 specifically affects the VAT Treg cell population and compromises organismal glucose metabolism (5, 7). Besides the transcription factors and cytokines, the structural component of the tissues such as mesenchymal stromal cells are known to imprint tissue specific features in Treg cells (8). While the tissue specific factors are critical, Foxp3 still plays a commanding and indispensable role in the Treg cell lineage (9).

This feature topic was commissioned to highlight recent advances in the field of Treg cells, particularly the emerging functions of Treg cells within and beyond the immune system. This special feature has two primary research articles and three reviews that emphasize the concept of Treg cell heterogeneity, from the point of development and function. Functional diversity of Treg cells is tightly linked to their location (3). This is clear from the non-canonical functions regulated by Treg cells that reside in organs such as VAT, colon, brain, and muscle (3). The gastrointestinal tract is a dynamic organ that is constantly exposed to environmental antigens (3, 4). Cosovanu and Neumann systematically review the literature to breakdown the developmental and functional heterogeneity of Treg cells in the gastrointestinal tract. Anatomically, Treg cells populate the small intestine, colon, and the Payer’s Patches to regulate distinct non-overlapping functions. The small intestine is predominantly populated by Gata3+ tTreg cells, which restrain inflammation and promote tissue repair to preserve gut homeostasis. The colon, however, is enriched for ROR*γ*t+ pTreg cells that are critical to maintaining tolerance to commensal microbiota. Besides these populations, a specialized population of Bcl6+ Treg cells localized in the germinal centers (GCs) regulate the production of IgA and further contribute to gut health. While several tissue-resident Treg cells were identified in the last few years, their cellular origin was poorly understood. Sivasami and Li review the landmark studies that gave birth to the ‘tissue Treg cell precursor’ concept in VAT Treg cells. This review discusses the definition and characteristics of tissue Treg cell precursors and the process by which they acquire tissue Treg cell features in a stepwise manner. Akin to tissue Treg cell precursors, two distinct precursor types for tTreg cells exist in the thymus. Santamaria et al. discuss the biology of Foxp3^lo^CD25^hi^ and Foxp3^hi^CD25^lo^ Treg precursors and mechanisms that drive their conversion to mature Treg cells with distinct functions.

While Treg cell heterogeneity is well studied in mouse models, emerging evidence also confirms the heterogeneity of Treg cells in humans (10). Matos et al. assessed 35 different functional markers using mass cytometry in cord blood and adult PBMCs to demonstrate Treg cell heterogeneity in humans. Their analysis revealed that cord blood Treg cells maintained a naive state but acquired an effector memory phenotype and heterogeneity upon ageing. They further show that Treg cell heterogeneity increased in the setting of allogenic stem cell transplantation by decreasing in chronic Graft *vs* host disease (GVHD), suggesting the dynamic population level changes in human Treg cells that track with age and disease. Under inflammatory conditions, Treg cells are known to lose their suppressive function and acquire an inflammatory phenotype. Chen et al. analyzed the phenotype of Treg cells in idiopathic orbital inflammation (IOI) and vision disorder. They found expansion of IL-17 producing Treg cells in the circulation of IOI patients and T_H_2 polarized Treg cells in the orbit. Circulating Treg cells in IOI patients had reduced expression of the IL-33 receptor ST2, which coincided with loss of IL-33 in orbital tissues. Exposing Treg cells from IOI patients to IL-33 reverted their inflammatory phenotype and restored the suppressive function.

Overall, this Research Topic features a collection of articles that provide a balanced portrayal of Treg cell heterogeneity in mice and humans from the perspective of development and function. There is, however, much to learn about the heterogeneity of Treg cells. Particularly, mechanisms that underpin context specific gene expression in Treg cells and the interaction between Treg cells and the tissue microenvironment in shaping heterogeneity remain to be studied.

## Author Contributions

ES, SH and AV edited this Research Topic and contributed to the drafting of editorial. All authors contributed to the article and approved the submitted version.

## Conflict of Interest

The authors declare that the research was conducted in the absence of any commercial or financial relationships that could be construed as a potential conflict of interest.

## Publisher’s Note

All claims expressed in this article are solely those of the authors and do not necessarily represent those of their affiliated organizations, or those of the publisher, the editors and the reviewers. Any product that may be evaluated in this article, or claim that may be made by its manufacturer, is not guaranteed or endorsed by the publisher.

## References

[1] Josefowicz SZ Lu LF Rudensky AY. Regulatory T Cells: Mechanisms of Differentiation and Function. Annu Rev Immunol (2012) 30:531–64. doi: 10.1146/annurev.immunol.25.022106.141623 PMC6066374 22224781

[2] Vasanthakumar A Kallies A. The Regulatory T Cell: Jack-Of-All-Trades. Trends Immunol (2015) 36:756–8. doi: 10.1016/j.it.2015.10.002 26511762

[3] Burzyn D Benoist C Mathis D. Regulatory T Cells in Nonlymphoid Tissues. Nat Immunol (2013) 14:1007–13. doi: 10.1038/ni.2683 PMC4708287 24048122

[4] Sefik E Geva-Zatorsky N Oh S Konnikova L Zemmour D McGuire AM. MUCOSAL IMMUNOLOGY. Individual Intestinal Symbionts Induce a Distinct Population of RORgamma(+) Regulatory T Cells. Science (2015) 349:993–7. doi: 10.1126/science.aaa9420 PMC4700932 26272906

[5] Cipolletta D Feuerer M Li A Kamei N Lee J Shoelson SE. PPAR-Gamma Is a Major Driver of the Accumulation and Phenotype of Adipose Tissue Treg Cells. Nature (2012) 486:549–53. doi: 10.1038/nature11132 PMC3387339 22722857

[6] Neumann C Blume J Roy U Teh P Vasanthakumar A Beller A. C-Maf-Dependent Treg Cell Control of Intestinal TH17 Cells and IgA Establishes Host-Microbiota Homeostasis. Nat Immunol (2019) 20:471–81. doi: 10.1038/s41590-019-0316-2 30778241

[7] Vasanthakumar A Moro K Xin A Liao Y Gloury R Kawamoto S. The Transcriptional Regulators IRF4, BATF and IL-33 Orchestrate Development and Maintenance of Adipose Tissue-Resident Regulatory T Cells. Nat Immunol (2015) 16:276–85. doi: 10.1038/ni.3085 25599561

[8] Vasanthakumar A Chisanga D Blume J Gloury R Britt K Henstridge D. Sex-Specific Adipose Tissue Imprinting of Regulatory T Cells. Nature (2020) 579:581–5. doi: 10.1038/s41586-020-2040-3 PMC7241647 32103173

[9] Hayatsu N Miyao T Tachibana M Murakami R Kimura A Kato T. Analyses of a Mutant Foxp3 Allele Reveal BATF as a Critical Transcription Factor in the Differentiation and Accumulation of Tissue Regulatory T Cells. Immunity (2017) 47:268–83.e269. doi: 10.1016/j.immuni.2017.07.008 28778586

[10] Wing JB Tanaka A Sakaguchi S. Human FOXP3(+) Regulatory T Cell Heterogeneity and Function in Autoimmunity and Cancer. Immunity (2019) 50:302–16. doi: 10.1016/j.immuni.2019.01.020 30784578

